# N6-methyladenosine RNA methylation regulators contribute to the progression of prostate cancer

**DOI:** 10.7150/jca.46379

**Published:** 2021-01-01

**Authors:** Qunying Wu, Xing Xie, Yiming Huang, Shanshan Meng, Youcheng Li, Huifeng Wang, Yanling Hu

**Affiliations:** 1 Department of Biochemistry and Molecular Biology, School of Pre-Clinical Medicine, Guangxi Medical University, Nanning, Guangxi, 530021, China.; 2 Life Sciences Institute, Guangxi Medical University, Nanning, Guangxi, 530021, China.; 3 Center of Genomic and Personalized Medicine, Guangxi Medical University, Nanning, Guangxi, 530021, China.; 4 Department of Clinical Laboratory, Guigang City People's Hospital, The Eighth Affiliated Hospital of Guangxi Medical University, Guigang, Guangxi, 537100, China.

**Keywords:** prostate cancer, N6-methyladenosine, castration-resistant prostate cancer, tumorigenesis and progression, androgen receptor

## Abstract

Prostate cancer (PCa) is one of the most common epithelial malignant tumors and the fifth leading cause of cancer death in men. An increasing number of studies have demonstrated that N6-methyladenosine (m^6^A) plays a crucial role in tumorigenesis and tumor development. However, little is known about the role and levels of common m^6^A regulators and m^6^A levels in PCa. In this study, we analyzed the characteristic expression of m^6^A regulators in PCa and castration-resistant prostate cancer (CRPC). UALCAN and cBioPortal were used to estimate the clinical value and genetic alterations of m^6^A regulators, respectively. The correlation between m^6^A regulators and androgen receptor (AR) was assessed using Gene Expression Profiling Interactive Analysis (GEPIA) by Pearson correlation statistics. Total m^6^A levels were detected in transgenic adenocarcinoma of the mouse prostate (TRAMP) mice and PCa cell lines. Results showed that the expression of methyltransferase-like 3 (METTL3) and YTH domain family members, namely, YTHDC2, YTHDF1, and YTHDF2 were generally upregulated in PCa, whereas those of fat mass and obesity-associated protein (FTO), AlkB homolog 5 (ALKBH5), and methyltransferase-like 14 (METTL14) were downregulated. The expression of METTL3, METTL14, Wilms' tumor 1-associating protein (WTAP), YTHDC2, YTHDF1, and YTHDF2 were remarkably higher in CRPC with lymph node metastasis than that in CRPC with bone metastasis, whereas ALKBH5, FTO, and YTHDF3 significantly decreased in CRPC with lymph node metastasis tissues. YTHDF1, YTHDF2, and YTHDC2 were positively correlated with the Gleason grades of PCa, and METTL14, FTO, and ALKBH5 were negatively associated with the Gleason classification. M^6^A regulators were positively correlated with AR. Patients with a genomic alteration of m^6^A were associated with poor disease-free survival (DFS). The total m^6^A levels in TRAMP mice increased dramatically compared with those in tumor-free mice, and m^6^A levels in LNCaP cell lines were higher than DU145 and PC3 cell lines. In summary, METTL3, METTL14, ALKBH5, FTO, YTHDC2, YTHDF1, and YTHDF2 were abnormally expressed in PCa and related to Gleason classification. Changes in m^6^A levels maybe contributed to the development and progression of PCa.

## Introduction

More than 160 posttranscriptional modifications of cellular RNAs have been discovered 1, and N6-methyladenosine (m^6^A) is the most prevalent RNA modification of mRNA and non-coding RNA in eukaryotic cells 2. M^6^A methylation regulators include methyltransferase complex, RNA demethylases, and specific RNA-binding proteins, which are also known as “writers,” “erasers,” and “readers,” respectively 3. Methyltransferase-like 3 (METTL3), methyltransferase-like 14 (METTL14), and Wilms' tumor 1-associating protein (WTAP) act as writers in mammalian cells 4, 5. AlkB homolog 5 (ALKBH5) and fat mass and obesity-associated protein (FTO) are m^6^A demethylases that mediate the reversal of m^6^A methylation 6, 7. The RNA modifications of m^6^A are recognized by “reader” proteins. These proteins are the YTH domain family members, including YTHDC1, YTHDC2, YTHDF1, YTHDF2, and YTHDF3 8. Growing evidence demonstrates that the aberrant expression of m^6^A RNA methylation is closely related to various cancers, such as hepatocellular carcinoma (HCC) 9, gastric cancer (GC) 10, 11, endometrial cancer 12, and glioblastoma 13. Targeting FTO/m^6^A/MYC/CEBPA signaling exerts an anti-tumor effect in most leukemia samples 14. The knockout of METTL14 substantially decreases the growth of Epstein-Barr virus-associated tumorigenesis in a xenograft model 15. These studies indicate that m^6^A modification plays a critical role in cancer and maybe a potential target for cancer therapy.

Prostate cancer (PCa) is one of the most common epithelial malignant tumors in aging males worldwide and the fifth leading cause of cancer death in men 16, 17. Androgen deprivation therapy (ADT) is the primary treatment for patients with advanced PCa, however, most patients eventually develop resistance to castration-resistant prostate cancer (CRPC) 18. Despite the emergence of new therapeutic approaches, patients with metastatic PCa have a low quality of life and a low 5-year survival rate of less than 30% 19, 20. The high morbidity and mortality of PCa necessitate more work to reveal its underlying mechanisms. Recent evidence suggests that m^6^A modification is closely connected to PCa. VIRMA and YTHDF3 are increased in stages III/IV than in stage II in PCa 21. Li *et al*. 22 found that YTHDF2 is highly expressed in PCa and the downregulation of YTHDF2 considerably increases the level of m^6^A and inhibits the proliferation and migration of PCa cell lines. Cai *et al*. 23 reported that the silencing of METTL3 by shRNA inhibits cell proliferation, colony formation, and invasion of PCa cells and inhibits the growth of tumors *in vivo*. However, little is known about the role of different m^6^A regulators in the development and progression of PCa. In this study, we evaluated the expression of m^6^A regulators, the correlation between m^6^A regulators and clinical outcomes, and the genetic mutations of these regulators in PCa. We also analyzed the role of m^6^A levels in PCa by *in vivo* and *in vitro* experiments.

## Materials and methods

### Expression data sets

The Cancer Genome Atlas (TCGA)-Prostate adenocarcinoma (TCGA-PRAD) cohort data and the gene expression of 495 patients with PCa (499 prostate tissues) and 52 normal samples were obtained from the National Cancer Institute Genomic Data Commons (https://portal.gdc.cancer.gov/). R version 3.6.3 software was utilized to normalize and process the expression data. Oncomine database (https://www.oncomine.org/) is a powerful data-mining platform that contains 715 independent datasets and the data of 86,733 tumor tissues and adjacent normal tissues 24. In this study, we analyzed the mRNA expression levels of m^6^A regulators in different types of cancer by Oncomine data mining. The search parameters were as follows: type of analysis: cancer versus normal tissues; type of data: mRNA; *P* value: 0.05. The Human Cancer Metastasis Database (HCMDB, http://hcmdb.i-sanger.com/index) 25 is an online integrated database for storing and analyzing the large-scale expression data of cancer metastasis. We selected the data set of EXP00339 in HCMDB (dataset ID: GSE74685), which contains 89 CRPC samples that comprise 20 samples of bone metastasis and 69 of lymph node metastasis, to explore the expression of m^6^A regulators in CRPC with different metastasis sites.

### M^6^A regulators and Gleason classification

UALCAN (http://ualcan.path.uab.edu) is an interactive online portal providing the gene expression and clinical analysis of tumor subsets, using TCGA level 3 RNA sequencing and clinical data from 31 cancer types 26. UALCAN was implemented to analyze the relative expression of m^6^A regulators in different Gleason classifications to understand the clinical importance of m^6^A regulators in PCa.

### M^6^A regulators and androgen receptor (AR)

The Gene Expression Profiling Interactive Analysis (GEPIA) database (http://gepia.cancer-pku.cn/index.html) 27 is a website for TCGA cancer and the Genotype-Tissue Expression profile analysis and interaction analysis. GEPIA can be used to perform the correlation analysis of gene pairs by Spearman, Pearson, or Kendall correlation statistics. The correlation between m^6^A regulators and AR was analyzed using GEPIA by Pearson correlation statistics.

### Genetic alteration of m^6^A regulators in PCa

CBioPortal (http://www.cbioportal.org/) is an open-access website that explores, visualizes, and analyzes multidimensional cancer genomics data. We selected eight studies (Prostate Adenocarcinoma [Broad/Cornell, Nat Genet 2012], Prostate Adenocarcinoma [Fred Hutchinson CRC, Nat Med 2016], Prostate Adenocarcinoma [MSKCC, PNAS 2014], Prostate Adenocarcinoma [TCGA, Firehose Legacy], Prostate Adenocarcinoma [CPC-GENE, Nature 2017], Prostate Adenocarcinoma [MSKCC, Cancer Cell 2010], Prostate Adenocarcinoma [MSKCC/DFCI, Nature Genetics 2018], and Prostate Cancer [DKFZ, Cancer Cell 2018]) to analyze the genetic alterations of m^6^A regulators in PCa. Disease-free survival (DFS) curve was drawn by the Kaplan-Meier method, and the difference in DFS was evaluated by log-rank test.

### Cell lines and culture conditions

Three human PCa cell lines were used in this study: PC3, DU145, and LNCaP. PC3 and DU145 cell lines were obtained from the Suzhou Institute of Biomedical Engineering and Technology, Chinese Academy of Sciences. LNCaP cell line was acquired from the Cell Bank of the Chinese Academy of Sciences (Shanghai, China). The basal medium was RPMI-1640 medium (Gibco) supplemented with 10% (v/v) fetal bovine serum (Gibco), 1% penicillin and 1% streptomycin (Invitrogen). LNCaP cells were cultured in basic medium supplemented with 1.0 mM L-glutamine and 1.0 mM sodium pyruvate (Invitrogen). All cells were incubated in a humidified incubator at 37°C and 5% CO_2_ (Thermo Scientific).

### TRAMP mice

Hemizygous male transgenic adenocarcinoma of the mouse prostate (TRAMP) mice (8247 Ng/J) and wild-type C57BL/6 females were obtained from the Model Animal Research Center of Nanjing University (MARCNU, Nanjing, Jiangsu, China). The mice were reared and propagated by the MARCNU protocol. Male offspring were genotyped by polymerase chain reaction (PCR) according to protocol. All animal experiments were performed following institutional guidelines and approved by the Institutional Animal Care and Use Committee of Guangxi Medical University.

### Detection of total m^6^A levels in TRAMP mice and PCa cell lines

Twelve TRAMP mice were randomly divided into four groups with three mice in each group. The prostate tissues of the mice at 8, 16, 26, and 36 weeks were collected for RNA extraction. Total RNA was extracted from the prostate tissues and PCa cell lines by TRIzol Reagent (Thermo Fisher Scientific). EpiQuik m^6^A RNA Methylation Quantification Kit (EpiGentek) was used to detect the total m^6^A RNA methylation levels.

### Statistical analysis

Statistical data were analyzed using GraphPad Prism version 7.0 (GraphPad Software, USA). An independent t-test was used to evaluate the expression of m^6^A regulators between PCa tissues and normal tissues, and the results were presented as the mean ± SD. Differences were considered statistically significant if* P* <0.05.

## Results

### M^6^A regulators in PCa were distinct from normal tissues

**Figure 1:**
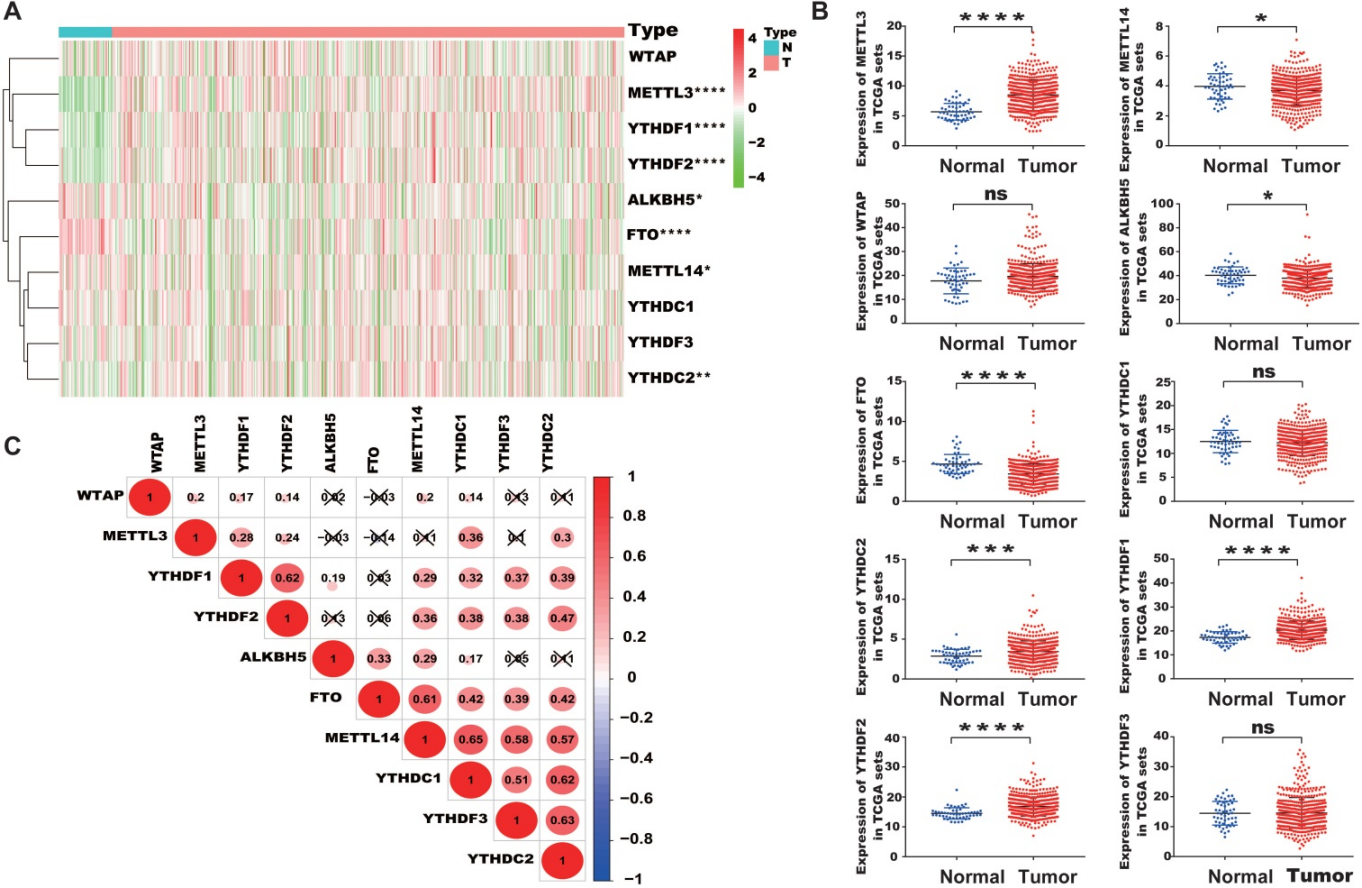
** Characteristic expression of m^6^A regulators in PCa.** (A) Heatmap of the expression of different m^6^A regulators between PCa tissues and normal tissue samples. (B) Scatter plots of the expression of m^6^A regulators in PCa. (C) Spearman correlation analysis of m^6^A regulators in PCa (Red and green colors in the plots symbolized relatively high and low expression, respectively).

We extracted the mRNA expression matrix of 10 common m^6^A regulators from TCGA-PRAD to evaluate their expression in PCa. The results showed that METTL3, a chief component of m^6^A methyltransferase, was remarkably increased in PCa tissues than in normal controls (*P* < 0.05, Figure 1A, 1B). YTHDC2, YTHDF1, and YTHDF2 were also substantially higher in PCa tissues than in normal controls, whereas the expression of FTO, ALKBH5, and METTL14 decreased in PCa. Then, the correlation among m^6^A regulators was analyzed. Results indicated that METTL14 was positively correlated with YTHDC1, YTHDF2, and YTHDC2, and YTHDC1, YTHDC2, YTHDF2, and YTHDF1 were positively correlated with each other (Figure 1C).

**Figure 2:**
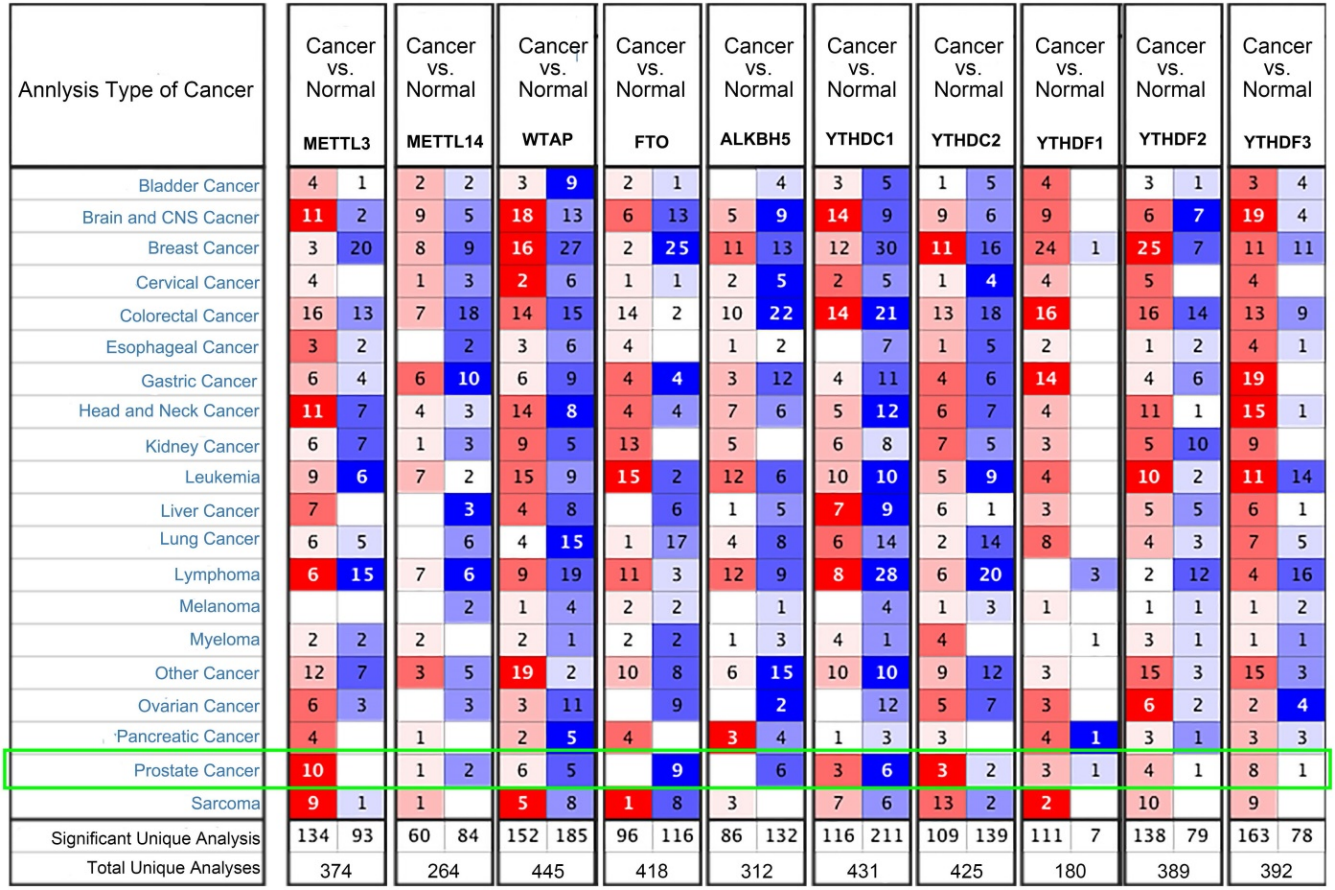
** Transcriptional levels of m^6^A regulators in PCa (Oncomine).** Arabic numbers in the figure showed the numbers of datasets in which m^6^A regulator expression was significantly upregulated (red) or downregulated (blue) in PCa tissues compared with adjacent normal tissues.

We examined the mRNA levels of m^6^A regulators in different PCa datasets based on the Oncomine database to confirm the expression patterns of the m^6^A regulators in PCa. The results showed that the expression trends of the 10 common m^6^A regulators were consistent with the TCGA-PRAD (Figure 2). METTL3 was highly expressed in 10 PCa datasets, and FTO and ALKBH5 were downregulated in nine and six datasets, respectively.

### Differential expression profiles of m^6^A regulators in CRPC

**Figure 3:**
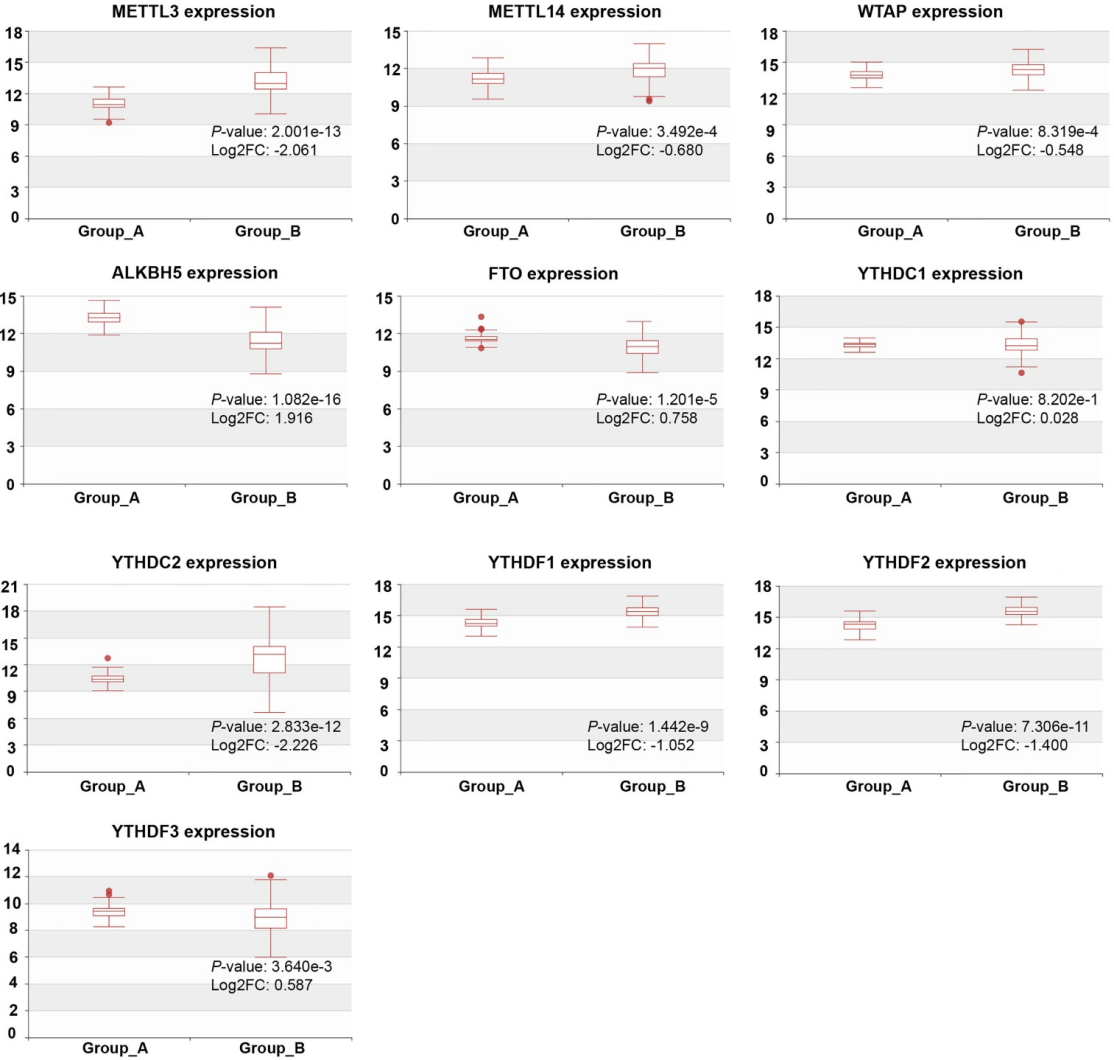
** Expression of m^6^A regulators in CRPC with different metastasis sites.** Group_A, metastasis tumor of CRPC with bone metastasis; Group_B, metastasis tumor of CRPC with lymph node metastasis experiment.

We detected the mRNA levels of m^6^A regulators in CRPC with different metastasis sites using HCMDB to further examine their expression in PCa. The results showed that, except YTHDC1, the expression of m^6^A regulators was statistically significant at different CRPC metastasis sites (*P* < 0.01, Figure 3). The expression of METTL3, METTL14, WTAP, YTHDC2, YTHDF1, and YTHDF2 was remarkably higher in CRPC with lymph node metastasis than in CRPC with bone metastasis, whereas ALKBH5, FTO, and YTHDF3 were substantially decreased in CRPC with lymph node metastasis.

### Correlation between the expression of m^6^A regulators and Gleason classification

**Figure 4:**
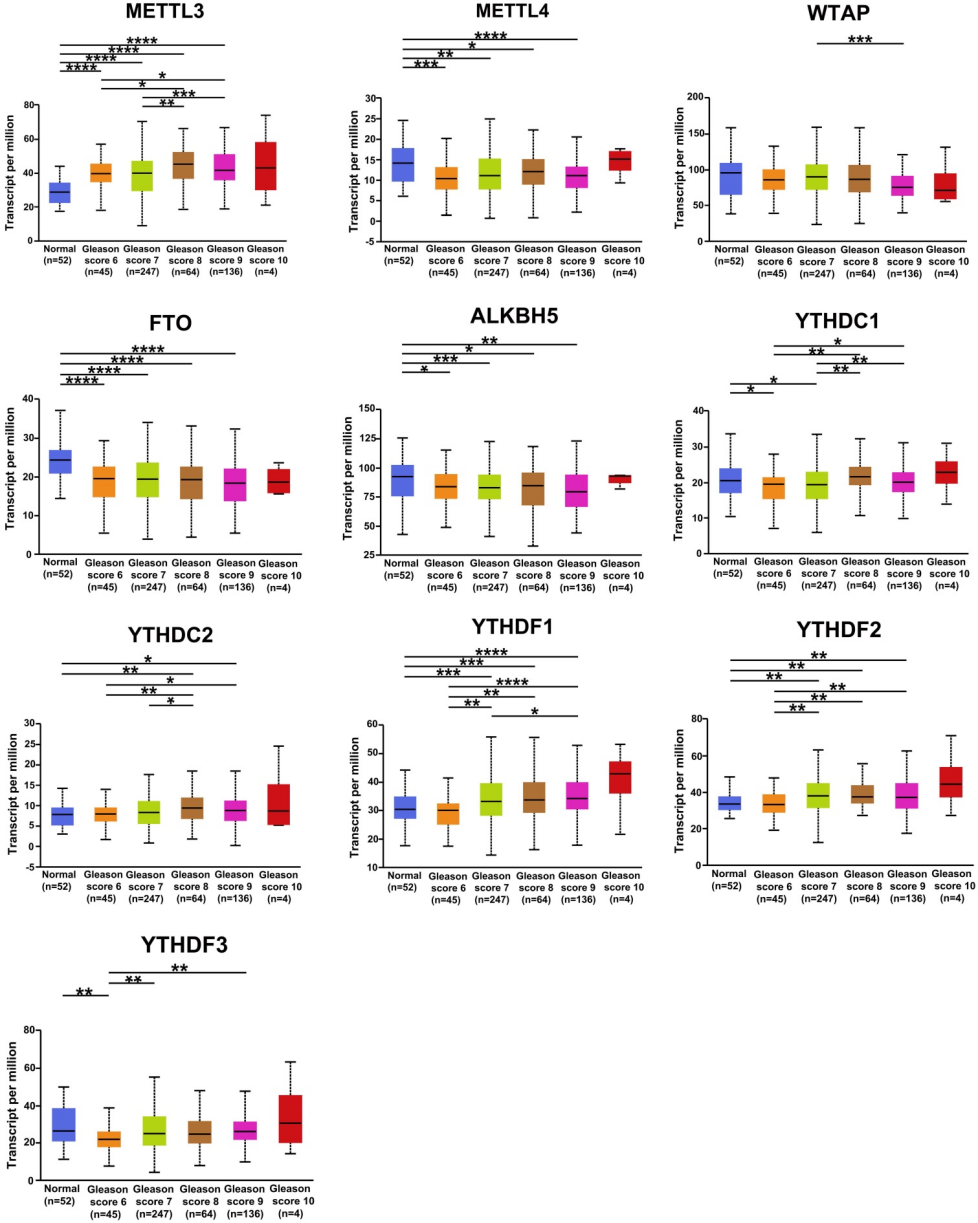
** Relative expression of m^6^A regulators in normal and PCa tissues with different Gleason classifications.** * P < 0.05, ** *P* < 0.01, ***P < 0.001.

The correlation between the expression of m^6^A regulators in PCa and Gleason classification was assessed using the UALCAN database. The results were shown in Figure 4. YTHDF1, YTHDF2, and YTHDC2 were positively correlated with Gleason grade, and the mRNA level of METTL3 was higher in patients with different Gleason grades of PCa than in normal samples. METTL14, FTO, and ALKBH5 were negatively associated with the Gleason classification of PCa. These results suggested that the mRNA expression of m^6^A regulators was related to the clinical features of patients with PCa.

### Correlation of m^6^A regulators and AR

**Figure 5:**
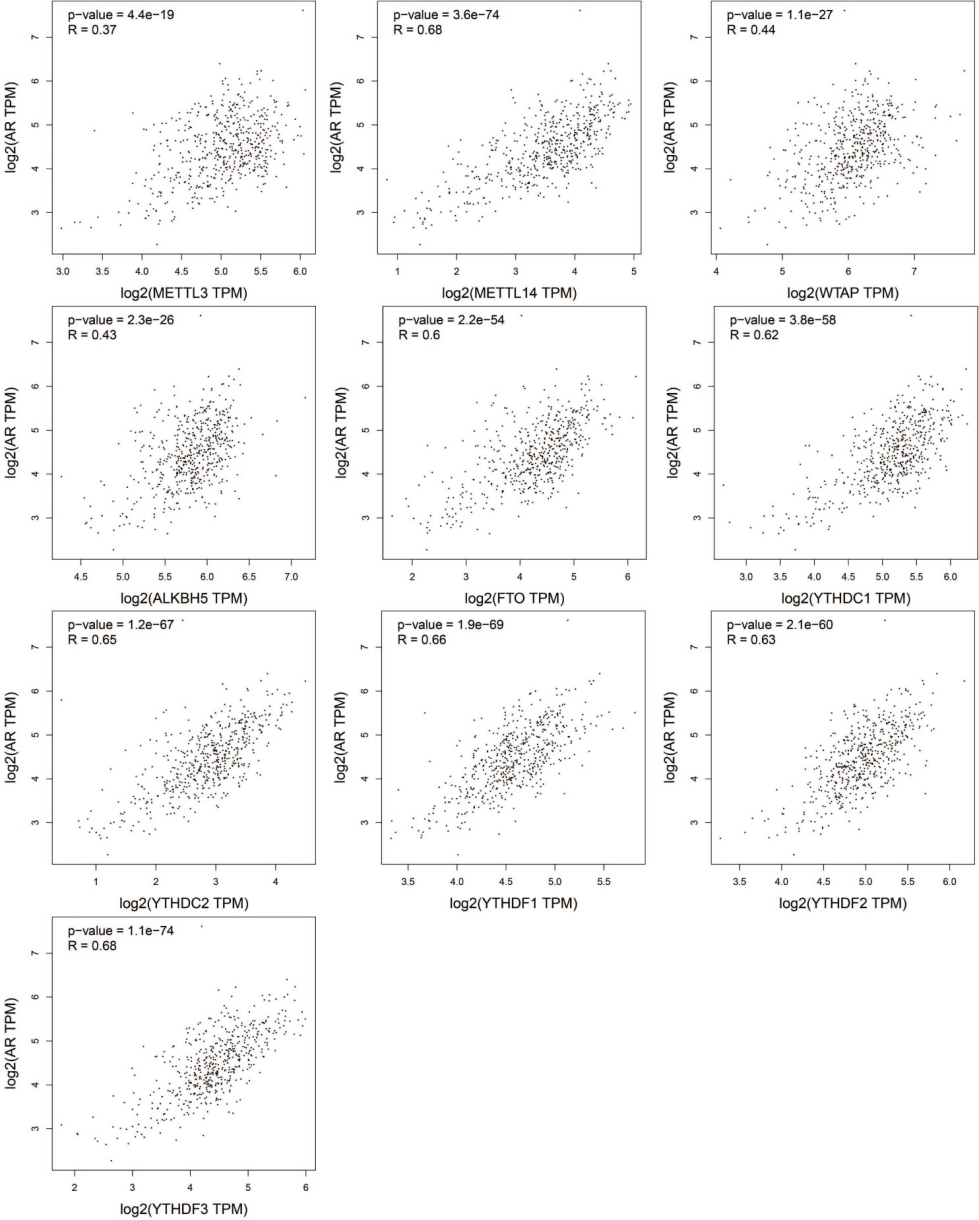
Correlation between the mRNA expression of m^6^A regulators and AR in PCa.

AR is a primary oncogene driver of PCa and plays a key role in the development and progression of PCa 28, 29. The progress of CRPC still depends on continuous AR signals 30. The relationship of the expression of m^6^A regulators and AR was assessed by GEPIA, and the results were presented in the scatter diagrams in Figure 5. Surprisingly, all the m^6^A regulators were positively correlated with AR (*P*<0.05). METTL14, FTO, YTHDC1, YTHDC2, YTHDF1, YTHDF2, and YTHDF3 were remarkably related to AR (*P* < 0.0001, R > 0.6).

### Genetic mutations of m^6^A regulators and their associations with the DFS of patients with PCa

**Figure 6:**
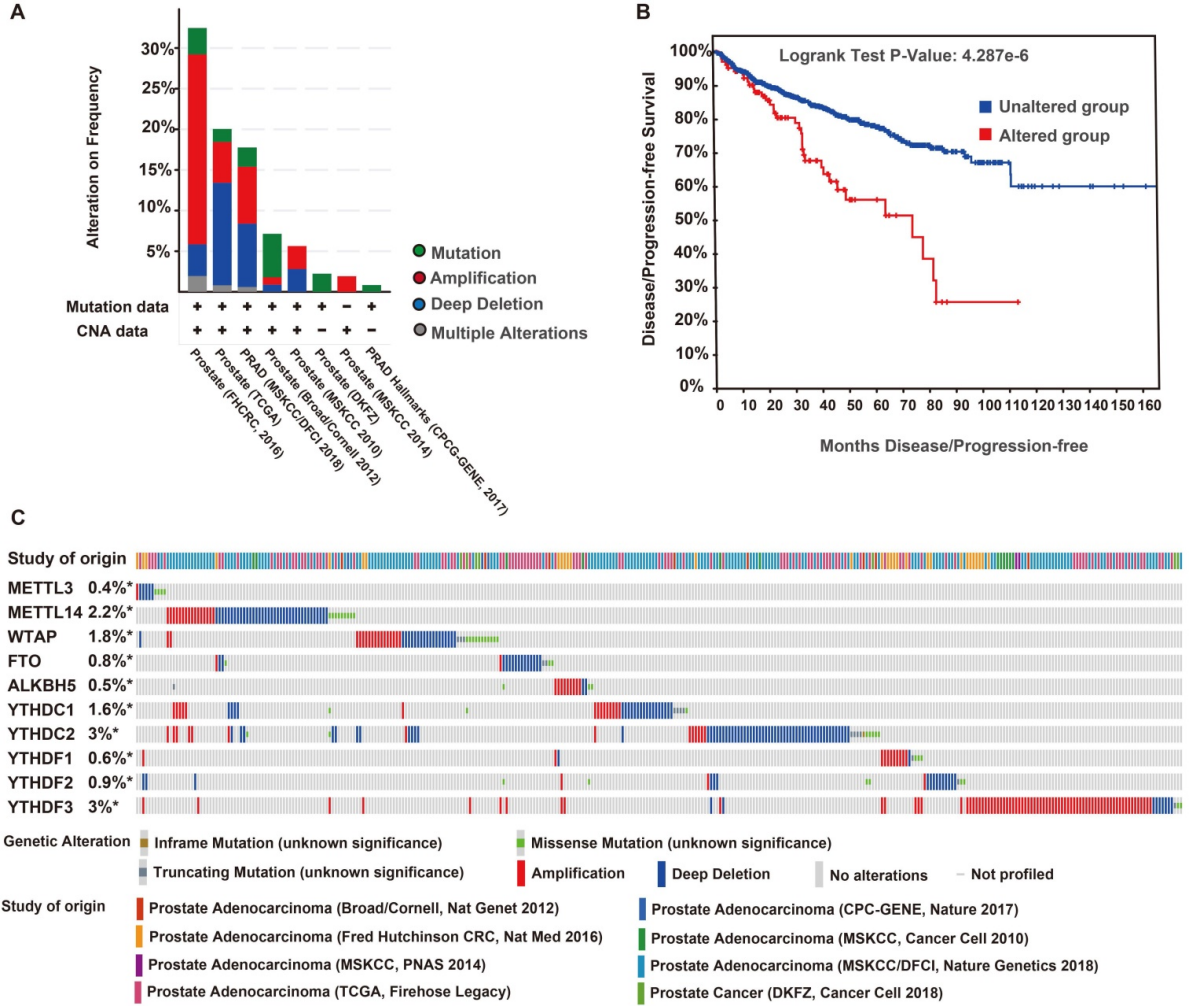
** Genomic alteration and prognosis of m^6^A regulators in eight PCa studies (cBioportal).** (A) OncoPrint of 10 m^6^A regulators in PCa. (B) DFS/ progression-free survival (PFS) of patients with PCa who had altered (red) and unaltered (blue) mRNA expression of the m^6^A regulators. (C) Genetic alteration frequencies of the 10 m^6^A regulators in PCa.

The genetic variations of the m^6^A regulators were analyzed using the cBioPortal database. We found 343 (12%) alterations from 2797 queried patients, including mutation, multiple alterations, amplification, and deep deletions. Deep deletion was the most common type of genomic alteration among these variations (Figure 6A). The DFS curves indicated that patients with a genomic alteration of m^6^A had remarkably worse DFS (Figure 6B). The alterations frequencies of YTHDC2, YTHDF3, METTL14, WTAP, YTHDC1, YTHDF2, FTO, YTHDF1, ALKBH5, and METTL3 were 3%, 3%, 2.2%, 1.8%, 1.6%, 0.9%, 0.8%, 0.6%, 0.5%, and 0.4%, respectively (Figure 6C).

### Expression of total m^6^A levels in TRAMP mice and PCa cell lines

**Figure 7:**
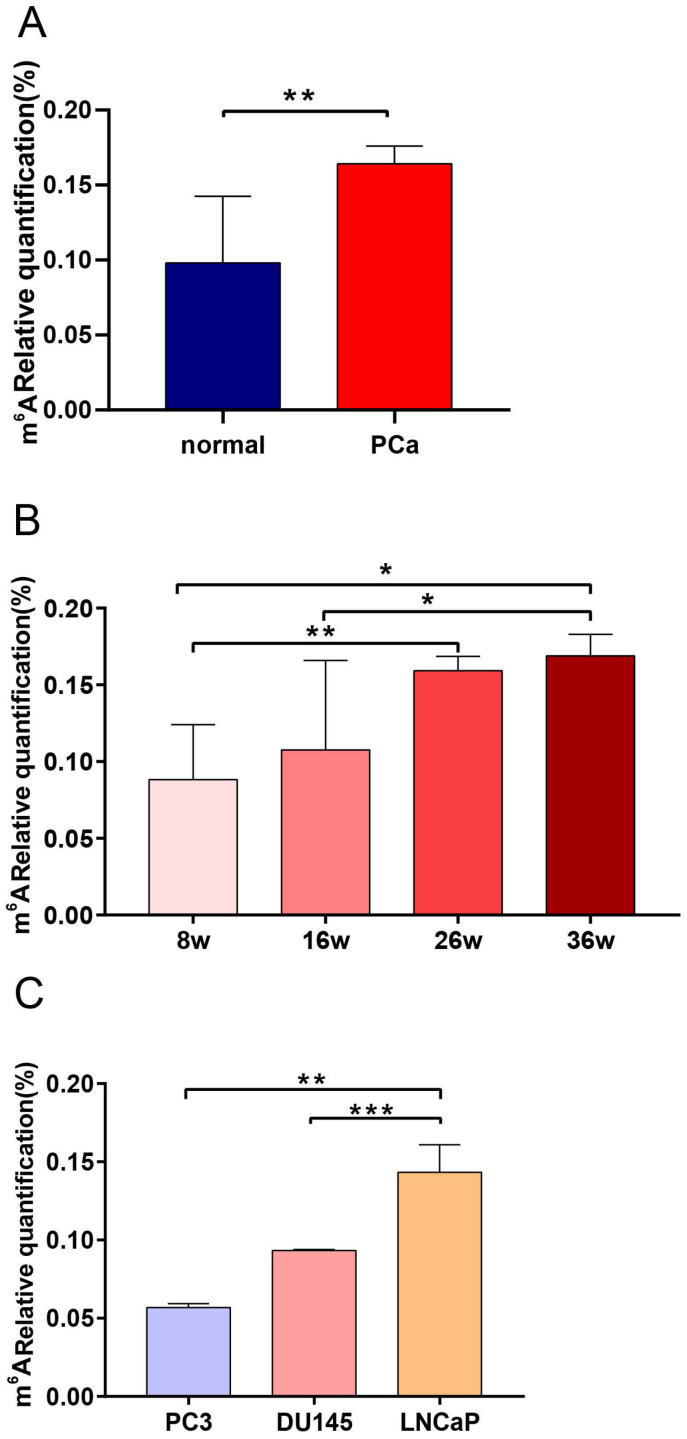
** Expression levels of the m^6^A in TRAMP mice and PCa cell lines.** (A) Expression of normal prostate tissues and PCa tissues in TRAMP mice. (B) Expression of m^6^A at different stages of TRMAP mice. (C) Expression of m^6^A in AR-independent and AR-dependent PCa cell lines.

TRAMP mice can spontaneously develop PCa and their pathological progression is similar to that of human PCa 31. We determined the total m^6^A expression of TRAMP mice at different stages of PCa to characterize the specific m^6^A level profile. Results showed that the m^6^A level of TRAMP mice increased significantly compared with that of tumor-free mice (*P* < 0.01; Figure 7A). We also found that m^6^A expression level was significantly upregulated with the increase in cancer progression (*P* < 0.05; Figure 7B). To evaluate the functional relevance of m^6^A between PCa and CRPC, we observed the m^6^A expression levels of AR-independent (DU145 and PC3) and AR-dependent PCa cell lines (LNCaP). The results showed that the m^6^A level of LNCaP cells increased significantly compared with those of DU145 and PC3 cells (Figure 7C).

## Discussion

M^6^A modification is involved in tumorigenesis, metastasis, and drug resistance 32. Wang *et al.* 10 found that increased expression of METTL3 in GC promotes tumor angiogenesis, supporting that METTL3 is a promising prognostic biomarker and therapeutic target for GC. It was reported that the downregulation of METTL14 is related to poor prognosis in patients with colorectal cancer (CRC), and the knockdown of METTL14 promotes the proliferation and invasion of CRC cells 33. Low expression of ALKBH5 was found to be closely linked to the occurrence and development of non-small cell lung cancer (NSCLC) 34. Elevated expression of some subtypes of FTO has a critical oncogenic role in acute myeloid leukemia (AML) 35. The upregulation of FTO promotes the development of melanoma and its resistance to PD-1 2. However, little is known about the role of multiple m^6^A regulators in PCa 23.

In this study, the expression, clinical value, and mutation of 10 m^6^A regulators in PCa, as well as their correlation with AR, were analyzed. Total m^6^A expression levels were determined in PCa cell lines and TRAMP mice. The results of our study showed that FTO and ALKBH5 had low- expression in PCa and were also significantly decreased in CRPC with bone metastasis tissues than in CRPC with lymph node metastasis. METTL3, YTHDC2, YTHDF1, and YTHDF2 had a remarkably high expression in PCa and CRPC with lymph node metastasis. METTL14 decreased in PCa and CRPC with bone metastasis tissues. The Gleason grading system is an effective predictor of the prognosis of patients with PCa because it can evaluate the differentiation and malignancy of PCa 36. We assessed the relationship of m^6^A regulators and their Gleason classifications. We found that METTL3, YTHDF1, YTHDF2, and YTHDC2 were generally upregulated with different Gleason grades, whereas METTL14, FTO, and ALKBH5 had low expression in patients with different Gleason classifications of PCa. We also found that patients with a genomic alteration of m^6^A were associated with poor DFS. These findings suggest that METTL3, METTL14, ALKBH5, FTO, YTHDC2, YTHDF1, and YTHDF2 were abnormally expressed in PCa and related to Gleason classification, and these m^6^A modulators were the potential molecular targets for the diagnosis and treatment for PCa.

TRAMP mice developed varying degrees of prostate hyperplasia at 6-12 weeks, progressed to severe hyperplasia and adenocarcinoma at 18 weeks, and presented primary tumors at 24-30 weeks 37. M^6^A methylation level contributes to the progression of human cancer 38, 39. We hypothesized that changes in m^6^A level promoted the development of PCa, thus, we detected the m^6^A levels in TRAMP mice and PCa cell lines. We found that the m^6^A level of TRAMP mice increased with ages. These results indicated that changes in m^6^A levels were possibly involved in the occurrence and development of PCa.

Our study had some limitations. First, although we found that m^6^A regulators were abnormally expressed in PCa, the potential diagnostic and therapeutic effects were not evaluated, hence, further research is necessary to explore whether m^6^A regulators could be used as diagnostic markers or therapeutic targets in PCa. Second, we did not elucidate the molecular mechanisms of m^6^A regulators in PCa. The detailed mechanism of m^6^A regulators in PCa warrants further investigation.

In conclusion, we analyzed the expression of m^6^A regulators, their relationship with Gleason grades and AR, and their genomic variations in PCa, and detected m^6^A levels in TRAMP mice and PCa cell lines. The results indicated that the abnormal expression of m^6^A regulators and abnormal m^6^A levels contribute to the development of PCa. This study provides a further understanding of epigenetic RNA modification in PCa.

## Abbreviations

PCa: Prostate cancer
m^6^A: N6-methyladenosine
CRPC: castration-resistant prostate cancer
AR: androgen receptor
DFS: disease-free survival
TRAMP: transgenic adenocarcinoma of the mouse prostate
METTL3: Methyltransferase-like-3
METTL14: Methyltransferase-like-14
WTAP: Wilms tumor 1 associated protein
ALKBH5: AlkB homolog 5
FTO: fat mass and obesity-associated protein
HCC: hepatocellular carcinoma
GC: gastric cancer
ADT: Androgen deprivation therapy
NCI-GDC: National Cancer Institute Genomic Data Commons
HCMDB: Human Cancer Metastasis Database
GEPIA: Gene Expression Profiling Interactive Analysis
NSCLC: non-small cell lung cancer
AML: acute myeloid leukemia
OV: ovarian cancer
